# A Case of Ectrogenesis

**Published:** 1885-08

**Authors:** W. J. Burt

**Affiliations:** Sec. Texas State Med. Ass’n., Austin, Texas


### CASE OF ECTROGENESIS.

*By W. J. Burt, M. D., Sec. Tex. Med. Ass'n, Austin, Texas.*

For Daniel's Texas Medical Journal.

ON the 8th of March, 1885, Mrs. M. was delivered of a male child, weighing about 8½ pounds. Three years previously her first child was born—a healthy well-formed girl. She was a well-developed woman, 21 years of age, with a good family history, so far as I could learn. In this child there was a peculiar want of development, in several particulars, that makes it one of some interest. The child had no *ears*, and only small fleshy projections, about the size of a grain of corn, on the sides of the head, about the places the ears should have developed. There were no openings in the soft parts—no meatus—and no depressions in the temporal bone corresponding to the bony canal.

The inferior maxillary bone was developed from its articulations to the angles, of proper bony material, but the chin, or that portion, between the angles, was simply a small cartilaginous band, running directly from one side to the opposite, making the chin flat and broad. Underneath the tongue, and attached to it, was a second tongue, shorter and thicker than the first, and partly cleft on its inferior surface. This growth pushed the tongue proper up in the roof of the mouth. The hard palate was cleft, and there was neither uvula nor post-septum nares.

The phalanges and meta-carpus forming each thumb, were small cartilaginous projections growing from the meta-carpo-carpal articulations. There were also five fingers to each hand, the fifth ones, quite small, without nails, fleshy and growing at right angles from the metacarpo-phalangeal articulations of the little or fourth fingers.

The child could not nurse, and swallowed with great difficulty, but received enough nourishment to keep it alive to the present date, though now extremely emaciated.

There in no history of anything, *felt, seen or dreamed* about, in any way to account for this curious freak of nature.

